# Molecular Reactivity and Absorption Properties of Melanoidin Blue-G1 through Conceptual DFT

**DOI:** 10.3390/molecules23030559

**Published:** 2018-03-02

**Authors:** Juan Frau, Daniel Glossman-Mitnik

**Affiliations:** 1 Departament de Química, Universitat de les Illes Balears, 07122 Palma de Mallorca, Spain; juan.frau@uib.es; 2 Laboratorio Virtual NANOCOSMOS, Departamento de Medio Ambiente y Energía, Centro de Investigación en Materiales Avanzados, Miguel de Cervantes 120, Complejo Industrial Chihuahua, Chihuahua, 31136 Chih, Mexico

**Keywords:** melanoidins, Blue-G1, conceptual DFT, chemical reactivity, dual descriptor, Parr function, maximum absorption wavelength

## Abstract

This computational study presents the assessment of eleven density functionals that include CAM-B3LYP, LC-wPBE, M11, M11L, MN12L, MN12SX, N12, N12SX, wB97, wB97X and wB97XD related to the Def2TZVP basis sets together with the Solvation Model Density (SMD) solvation model in calculating the molecular properties and structure of the Blue-G1 intermediate melanoidin pigment. The chemical reactivity descriptors for the system are calculated via the conceptual Density Functional Theory (DFT). The choice of the active sites related to the nucleophilic, electrophilic, as well as radical attacks is made by linking them with the Fukui function indices, the electrophilic Parr functions and the condensed dual descriptor Δf(r). The prediction of the maximum absorption wavelength tends to be considerably accurate relative to its experimental value. The study found the MN12SX and N12SX density functionals to be the most appropriate density functionals in predicting the chemical reactivity of the studied molecule.

## 1. Introduction

In food science, the Maillard reaction is well known (named after the French Chemist who first described it): it consists of the reaction produced between a reducing sugar and an amino acid (the link of a small number of amino acids forms peptides, and at a higher number, they form proteins). When a reducing sugar reacts with a protein, a compound called a Schiff base is formed, and the accumulation of several of these compounds degrades proteins until they create Advanced Glycation End-products (AGEs) that originate fibrils that accumulate in the brain. It is hypothesized that when brain proteins degrade, they may cause degenerative diseases such as Alzheimer’s, Parkinson’s or diabetes.

The completion of the Maillard or nonenzymatic browning reaction leads to melanoidin formation. If reducing sugars are left to react with biological molecules under physiological conditions, a similar reaction occurs in a process referred to as glycation. Notably, glycation has a considerable influence on the aging process among living organisms. It also has an impact on the pathology of several diseases [1]. In recent times, our emphasis has been on understanding how glycation takes place and the chemical reactivity that reducing carbohydrates have with amino acids, as well as the peptides participating in the process, which are often linked to some diseases, such as Parkinson’s, diabetes and Alzheimer’s [2,3,4,5,6,7,8,9,10,11,12,13,14,15,16,17,18,19,20,21,22,23,24,25,26,27,28,29,30,31,32].

The main feature notable in the Maillard reaction includes color formation. However, little is known about the colored moieties that cause the coloration. The light-weight intermediate colored products are referred to as Colored Maillard Reaction Products (CMRP), and it is possible to measure color production based on absorbance readings that the visible region of the spectrum indicates. The typical wavelengths that apply in measurements include 360 and 420 nm [33]. The CMRPs attract interest from the food industry, as well as other industries because they are hypothesized as having the potential of being photosensitizers, in which the can be employed as antioxidants associated with health, as well as colorants that dye-sensitized solar cells can use to produce alternative energy. These potential applications explain the focus on their chemical properties, with their molecular reactivity experiencing particular interest. Blue-G1 is among the interesting CMRPs that have been studied using conceptual DFT [34]. As a blue pigment, it is isolated from the reaction system involving D-glucose and glycine. This prompts the assumption that Blue-G1 may be of interest in applying the concepts of density functional theory in studying the chemical reactivity that the blue pigment exhibits.

Conceptual Density Functional Theory (DFT), which is also called chemical reactivity theory [2,3,4,5,6,7,8,9,10,11,12,13,14,15,16,17,18,19,20,21,22,23,24,25,26,27,28,29,30,31,32], provides a powerful tool that can be used in predicting, analyzing and interpreting the outcomes that chemical reactions generate [35,36,37,38]. Parr et al. [35] pioneered the coverage of the topic. However, several useful concepts that followed their work result from the analysis done on the density of molecular systems using the DFT. The concepts enable researchers to qualitatively predict how the chemical reactivity will take place in a given system. It is also possible to quantify the concepts. Collectively, the concepts would be referred to as conceptual DFT descriptors.

A new theory applicable in studying reactivity in organic chemistry was presented by Luis R. Domingo [39]. The theory is known as Molecular Electron Density Theory (MEDT) and proposes that the capability of the changes in electron density affects the molecular reactivity [39], and the way electron density is distributed at the ground determines the physical, as well as the chemical molecular attributes. MEDT encompasses DFT and also defines a number of new concepts and reactivity descriptors including the nucleophilicity N index, the Global Electron Density Transfer (GEDT), as well as the local condensed descriptors like the electrophilic P${}_{k}^{+}$ and nucleophilic P${}_{k}^{-}$ Parr functions [40]. Gradually, Parr functions are growing in popularity in efforts to understand organic chemistry processes. Several recent publications on Diels–Alder, as well as cycloaddition reactions evidence the rising popularity [41,42,43].

The Kohn–Sham theory involves calculating the molecular density, the energy that the system has and the orbital energies, particularly those associated with frontier orbitals including HOMO and LUMO [44,45,46,47,48,49]. The theory would be necessary in establishing the quantitative values of the various conceptual DFT descriptors. Interest in using Range-Separated (RS) exchange correlation functionals in Kohn–Sham DFT has been on the rise recently [50]. The functionals tend to partition the r${}_{12}^{-1}$ operator and exchange them into long- and short-range parts, whose range separation parameter, ω, controls the rate of attaining the long-range behavior. It is possible to fix the value of ω. The value can also be “tuned” through a system-by-system mechanism that minimizes some tuning norm. The basis of the optimal tuning approach is the knowledge that the energy that the Highest Occupied Molecular Orbital (HOMO) should have, $\epsilon_{H}$(N), in exact Kohn-Sham (KS), as well as Generalized KS theory for an N electron system ought to be exactly −IP(N). Hereby, IP represents the vertical ionization potential that is calculated considering a particular functional, the energy difference E(N−1) − E(N). If approximate functionals are used, it would be possible to have considerable differences between $\epsilon_{H}$(N) and −IP(N). Optimal tuning constitutes determining a system-specific range-separation parameter ω non-empirically in an RSEfunctional. Optionally, it also implied that several other parameters including $\epsilon_{H}$(N) = −IP(N) are satisfied optimally [51]. Even though no equivalency exists to match this prescription of the Electron Affinity (EA) coupled with LUMO in the case of neutral species, it is possible that $\epsilon_{H}$(N + 1) = −EA(N), which facilitates the finding of an optimized value of ω that is then optimized to establish both properties. Such would make it easy to predict the conceptual DFT descriptors. The simultaneous prescription has in the past been referred to as the “KIDprocedure”, courtesy of the analogy it shares with Koopmans’ theorem [2,3,4,5,6,7,8,9,10,11,12,13,14,15,16,17,18,19,20,21,22,23,24,25,26,27,28,29,30,31,32].

This implies that the appropriateness that a particular density functional has in making a prediction on the conceptual DFT descriptors directly by relying on the property that the neutral molecule can be easily estimated. It only requires one to check the way it has followed the KID procedure. Nevertheless, tune optimization depends on the system and had to be performed for each molecule one at a time. Therefore, examining the various density functionals exhibiting significant accuracy across various types of databases in physics, chemistry, as well as where the ω value is fixed is done to determine how to perform the practical technique.

This study seeks to undertake a comparative study of the way a number of recent density functionals perform in reproducing the chemical reactivity descriptors that the Blue-G1 pigment has in the KID formalism to have sufficient insights about their molecular attributes that future studies use on the chemical reactivity that colored melanoidins of larger molecular weights that form from the reaction that reducing sugars have with proteins and peptides.

## 2. Theoretical Background

The theoretical background of this study is similar to the previous conducted research presented [2,3,4,5,6,7,8,9,10,11,12,13,14,15,16,17,18,19,20,21,22,23,24,25,26,27,28,29,30,31,32] and will be shown here to be complete, because this research is a component of a major project that is in progress.

If we consider the KID procedure presented in our previous works [2,3,4,5,6,7,8,9,10,11,12,13,14,15,16,17,18,19,20,21,22,23,24,25,26,27,28,29,30,31,32] together with a finite difference approximation, then the global reactivity descriptors can be written as [36,52,53,54,55] :
Electronegativity$\chi =-\frac{1}{2}\left(I+A\right)\approx \frac{1}{2}\left(\epsilon_{L}+\epsilon_{H}\right)$Global hardness$\eta =\left(I-A\right)\approx \left(\epsilon_{L}-\epsilon_{H}\right)$Electrophilicity$\omega =\frac{\mu^{2}}{2\eta }=\frac{{\left(I+A\right)}^{2}}{4(I-A)}\approx \frac{{\left(\epsilon_{L}+\epsilon_{H}\right)}^{2}}{4(\epsilon_{L}-\epsilon_{H})}$Electron-donating power$\omega^{-}=\frac{{\left(3I+A\right)}^{2}}{16(I-A)}\approx \frac{{\left(3\epsilon_{H}+\epsilon_{L}\right)}^{2}}{16\eta }$Electron-accepting power$\omega^{+}=\frac{{\left(I+3A\right)}^{2}}{16(I-A)}\approx \frac{{\left(\epsilon_{H}+3\epsilon_{L}\right)}^{2}}{16\eta }$Net electrophilicity$\Delta \omega^{\pm }=\omega^{+}-\left(-\omega^{-}\right)=\omega^{+}+\omega^{-}$
where $\epsilon_{H}$ and $\epsilon_{L}$ are the energies of the Highest Occupied and the Lowest Unoccupied Molecular Orbitals (HOMO and LUMO), respectively.

Applying the same ideas, the definitions for the local reactivity descriptors are [37,52,56,57,58,59,60,61,62,63]
 Nucleophilic Fukui function $f^{+}\left(r\right)=\rho_{N+1}\left(r\right)-\rho_{N}\left(r\right)$Electrophilic Fukui function$f^{-}\left(r\right)=\rho_{N}\left(r\right)-\rho_{N-1}\left(r\right)$Dual descriptor$\Delta f\left(r\right)={\left(\frac{\partial \;f(r)}{\partial \;N}\right)}_{\upsilon (r)}$Nucleophilic Parr function$P{}^{-}\left(r\right)=\rho_{s}^{rc}\left(r\right)$Electrophilic Parr function$P{}^{+}\left(r\right)=\rho_{s}^{ra}\left(r\right)$
where $\rho_{N+1}\left(r\right)$, $\rho_{N}\left(r\right)$ and $\rho_{N-1}\left(r\right)$ are the electronic densities at point r for the system with N+1, *N* and N−1 electrons, respectively, and $\rho_{s}^{rc}\left(r\right)$ and $\rho_{s}^{ra}(r$) are related to the Atomic Spin Density (ASD) at the **r** atom of the radical cation or anion of a given molecule, respectively [40].

## 3. Settings and Computational Methods

Consistent with the work presented earlier [2,3,4,5,6,7,8,9,10,11,12,13,14,15,16,17,18,19,20,21,22,23,24,25,26,27,28,29,30,31,32], this study involved performing computational studies with the Gaussian 09 series of programs [64] whose density functional methods are implemented in the computational package. The gradient technique was relied upon to determine the equilibrium geometries of the molecules; whereas the determination of the force constants and vibrational frequencies involves computing analytical frequencies on various stationary points that are obtained as optimization is completed to check whether the minima were real. The basis set that this work used included Def2SVP for both geometry optimization, as well as frequencies; whereas Def2TZVP was involved in calculating the electronic characteristics [65,66].

Eleven density functionals were selected to calculate the molecular structure and properties that the studied systems have. The selected functionals offer satisfactory results consistently in relation to a number of thermodynamic and structural attributes. Among the functionals are included CAM-B3LYP, which entails Handy and co-workers’ long-range-corrected version of B3LYP done through the Coulomb-attenuating method [67]. Another functional relates to LC-wPBEconstituting the long-range-corrected wPBE density functional [68]. In addition, M11entails a range-separated hybrid meta-Generalized Gradient Approximation (GGA) [69]. M11L, on the other hand, is a dual-range local meta-GGA [70], while MN12L consists of a non-separable local meta-Non-separable Gradient Approximation (NGA) [71]. MN12SX entails a range-separated hybrid non-separable meta-NGA [72], while N12 is an NGA [73]. N12SX comprise of a range separated hybrid NGA [72] as the wB97 and wB97X long-range corrected density functionals [74] together with the wB97XD version include empirical dispersion [75]. GGA in these functionals represents the generalized gradient approximation whereby the density functional is dependent on the up and down spin densities together with their reduced gradient; whereas NGA denotes the non-separable gradient approximation in which the density functional tends to rely on the up and down spin densities, as well as their lowered gradient, while also assuming a non-separable form. The various calculations were done with water being the solvent. Integral Equation Formalism-Polarized Continuum Model (IEF-PCM) computations were involved as the Solvation Model Density (SMD) provided [76].

## 4. Results and Discussion

This study took the molecular structure of the Blue-G1 intermediate melanoidin pigment from PubChem (https://pubchem.ncbi.nlm.nih.gov), a website that acts as the public repository for information pertaining to chemical substances together with the biological activities with which they are associated. The molecular structure with the IUPAC (International Union of Pure and Applied Chemistry) name includes 5-[1,4-bis-carboxymethyl-5-(2,3,4-trihydroxybutyl)-1,4-dihydropyrrolo[3,2-b]pyrrol-2-ylmethyl-ene]-1,4-bis-carboxymethyl-2-(2,3,4-trihydroxybutyl)-4,5-dihydropyrrolo[3,2-b]pyrrol-1-ium [34]. The pre-optimization of the resultant system involved selecting the most stable conformers. The selection was done using random sampling, which involved molecular mechanics techniques and inclusion of the various torsional angles via the general MMFF94force field [77,78,79,80,81], which involves the Marvin View 17.15 program, which constitutes an advanced chemical viewer suited to multiple and single chemical queries, structures and reactions (https://www.chemaxon.com). Afterwards, the structure that the resultant lower-energy conformer assumes was reoptimized using the eleven density functionals previously mentioned in the previous section together with the Def2SVP basis set, as well as the SMD solvation model in which the solvent was water.

The analysis of the results obtained in the study aimed at verifying that the KID procedure was fulfilled. Upon doing this previously, several descriptors associated with the results that HOMO and LUMO calculations obtained are related to the results obtained using the vertical I and A following the ΔSCFprocedure. A link exists between the three main descriptors and the simplest conformity to Koopmans’ theorem by linking $\epsilon_{H}$ with -I, $\epsilon_{L}$ with -A, and their behavior in describing the HOMO-LUMO gap as $J_{I}=\left|\epsilon_{H}+E_{gs}\left(N-1\right)-E_{gs}\left(N\right)\right|$, $J_{A}=\left|\epsilon_{L}+E_{gs}\left(N\right)-E_{gs}\left(N+1\right)\right|$ and $J_{HL}=\sqrt{{J_{I}}^{2}+{J_{A}}^{2}}$. Notably, the $J_{A}$ descriptor is an approximation that is only valid if the HOMO of the radical anion (the SOMO) shares similarity with the LUMO of the neutral system. Consequently, we decided to design another descriptor ΔSL, to guide in verifying how the approximation is accurate.

Table 1 illustrates the electronic energies of neutral, positive and negative molecular systems of Blue-G1, HOMO, LUMO and SOMO orbital energies (all in au), while the calculation of $J_{I}$, $J_{A}$, $J_{HL}$ and ΔSL descriptors involves using the eleven density functionals, as well as the Def2TZVP basis set that would use water as a solvent that is simulated through the SMD parametrization of the IEF-PCM model.

Afterwards, the study focuses on the other four descriptors analyzing the effectiveness of the density functional in predicting the electronegativity χ, the global electrophilicity ω, the global hardness η and for a combination of the conceptual DFT descriptors, taking into account the energies that the LUMO and HOMO or the vertical I and A are related; $J_{\chi }=\left|\chi -\chi_{K}\right|$, $J_{\eta }=\left|\eta -\eta_{K}\right|$, $J_{\omega }=\left|\omega -\omega_{K}\right|$, and $J_{CDFT}=\sqrt{J_{\chi }^{2}+J_{\eta }^{2}+J_{\omega }^{2}}$, where CDFT stands for Conceptual DFT.

Next, we consider four other descriptors that analyze how useful the studied density functionals are for the prediction of the electronegativity χ, the global hardness η, the global electrophilicity ω and for a combination of these conceptual DFT descriptors, considering only the energies of the HOMO and LUMO or the vertical I and A: $J_{\chi }=\left|\chi -\chi_{K}\right|$, $J_{\eta }=\left|\eta -\eta_{K}\right|$, $J_{\omega }=\left|\omega -\omega_{K}\right|$, and $J_{CDFT}=\sqrt{J_{\chi }^{2}+J_{\eta }^{2}+J_{\omega }^{2}}$, where CDFT stands for Conceptual DFT. The results of the calculations of $J_{\chi }$, $J_{\eta }$, $J_{\omega }$ and $J_{CDFT}$ for the Blue-G1 intermediate melanoidin pigment are displayed in Table 2.

As Table 1 provides, the KID procedure applies accurately for MN12SX and N12SX density functionals that are range-separated hybrid meta-NGA, as well as range-separated hybrid NGA density functionals, respectively. In fact, the values of J${}_{I}$, J${}_{A}$ and J${}_{HL}$ are actually not zero. Nevertheless, the results tend to be impressive especially for the MN12SXdensity functional. In addition, the ΔSL descriptor reaches the minimum values when the MN12SX and N12SX density functionals are used in the calculations. This implies that there are sufficient justifications to assume that the LUMO of the neutral approximates the electron affinity.

It can be seen that the same density functional follows the KID procedure in the rest of the descriptors such as $J_{\chi }$, $J_{\eta }$, $J_{\omega }$ and $J_{D1}$. The significance of these results is attributable to their illustration that reliance on $J_{I}$, $J_{A}$ and $J_{HL}$ would not be sufficient. For instance, if J${}_{\chi }$ were considered on its own to apply to each density functional covered in this study, the values would considerably be near zero. In the case of the other descriptors, only the MN12SX and N12SX density functionals exhibit this behavior. This implies that the results that J${}_{\chi }$ record are likely to be because of the cancellation of errors by chance.

The Blue-G1 intermediate melanoidin pigment would be better studied using the Time-Dependent Density Functional Theory (TDDFT) because the pigment is a colored molecule. Various TDDFT studies of different sizes of molecules have used the optimally-tuned RSH density functionals. The studies report that absorption properties improve especially when applying the charge-transfer phenomenon [51,82,83,84,85,86,87,88,89,90,91,92,93,94,95,96,97,98,99,100].

The considerable success of the approach is however undermined by the issue of tuning being system dependent. Therefore, focus should be on establishing the effectiveness of the behaviors of the fixed RSH density functionals in describing the excitation characteristics. In his works, Becke has recently mentioned that the adiabatic connection and the ideas of Hohenberg, Kohn and Sham apply only to electronic ground states comprise a common misconception [101]. Furthermore, consistent with Baerends et al., the KS model is not appreciated for being superior because of its lowest excitation energy in molecules. Physically, it amounts to an excitation of the KS system rather than electron addition, as would be the case in Hartree–Fock. Thus, it can effectively be used as a measure of the optical gap and is an effective approximation to the gap (in molecules) [102]. In their conclusion, van Meer et al. advance that the HOMO-LUMO gap associated with the KS model tends to be an approximation of the lowest excitation energy, a desirable characteristic with no concerns regarding it [103].

**Figure 1:**
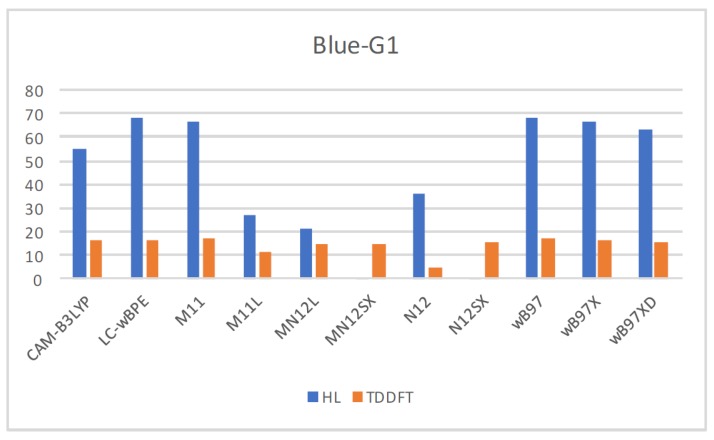
A graphical comparison of the results for the calculation of the $\lambda_{max}$ of the Blue-G1 pigment between the HOMO-LUMO gap prediction, the TDDFT values and the experimental data.

Therefore, calculation of the maximum wavelength absorption of the Blue-G1 pigment involved conducting TDDFT calculations with the aforementioned eleven density functionals at the same level of model chemistry and theory as the calculations for establishing the ground state of the molecule. Table S1 of the Supplementary Information presents the results by comparing the values involved in the ground-state approximation derived from the HOMO-LUMO gap, as well as the TDDFT ones together with the experimental value of 629 nm [34]. Moreover, Figure 1 provides an illustration that compares the results graphically.

Notably, the presented results suggest that the differences with the experimental value for $\lambda_{max}$ tend to have the same order in the various functionals that the current study considers, apart from the N12 density functional. If the $\lambda_{max}$ values that the HOMO-LUMO gap generates were the ones considered, MN12SX and N12SX would appear to be accurate especially in predicting this value. This does not apply in the rest of the density functionals that the study considers. In fact, the results that the TDDFT calculations give can be improved by expanding the basis set and integrating vibronic corrections; despite the level of accuracy in predicting the excited state property being remarkable if calculations are made at the ground state with the suitable choice of density functional.

**Figure 2:**
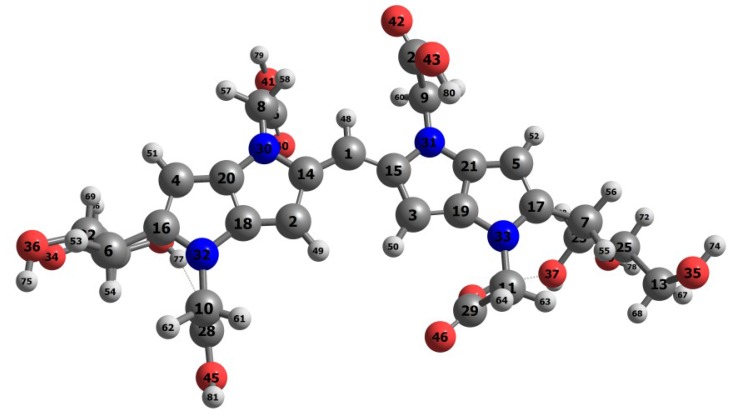
An schematic representation of the optimized structure of the Blue-G1 pigment calculated with the MN12SX density functional showing the numbering of the atoms.

Upon verifying that the MN12SX density functional is the best suited for the calculation of the global reactivity density descriptors and in predicting the $\lambda_{max}$ in agreement with the experimental, Figure 2 presents the optimized structure o the Blue-G1 pigment graphically as calculated based on the theory; whereas Tables S2 and S3 of the Supplementary Materials illustrate the bond length and the bond angles.

Table 3 illustrates the results obtained after calculating the electronegativity χ, chemical hardness η, global electrophilicity ω, electron-accepting ($\omega^{+}$) and electron-donating ($\omega^{-}$) powers, as well as net electrophilicity powers with the MN12SX density. The Def2TZVP basis set is used with water acting as a solvent in line with the SMD solvation model.

The calculations of the condensed Fukui functions and dual descriptor are done by using the Chemcraft molecular analysis program to extract the Mulliken and NPAatomic charges [104] beginning with single-point energy calculations involving the MN12SX density functional that uses the Def2TZVP basis set. In line with the SMD solvation model, water is utilized as a solvent.

**Figure 3:**
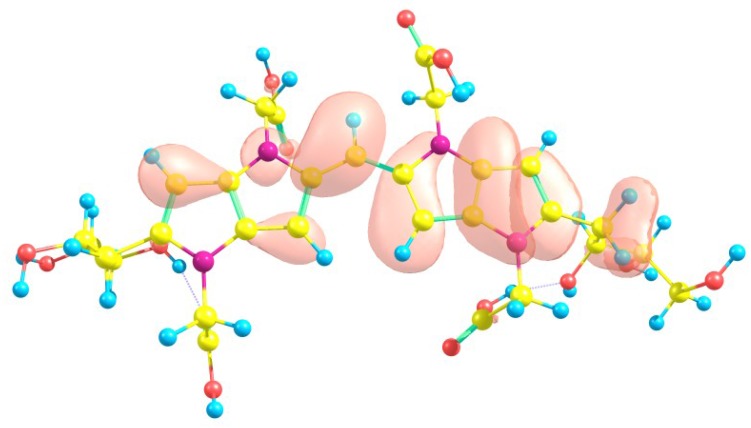
A graphical schematic representation of the radical Fukui function $f^{0}$ of the Blue-G1 intermediate melanoidin pigment.

Considering the potential application of the Blue-G1 molecule as an antioxidant, it is of interest to get insight into the active sites for radical attack. A graphical representation of the radical Fukui function $f^{0}$ is presented in Figure 3.

The condensed electrophilic and nucleophilic Parr functions *P*${}_{k}^{+}$ and *P*${}_{k}^{-}$ over the atoms of the Blue-G1 pigment (excluding the H atoms) have been calculated by extracting the Mulliken and Hirshfeld (or CM5) atomic charges using the Chemcraft molecular analysis program [104] starting from single-point energy calculations of the ionic species with the MN12SX density functional using the Def2TZVP basis set in the presence of water as a solvent according to the (SMD) solvation model.

The results for the condensed dual descriptor calculated with Mulliken atomic charges Δf${}_{k}$ (M), with NPA atomic charges Δf${}_{k}$ (N), the electrophilic and nucleophilic Parr functions with Mulliken atomic charges *P*${}_{k}^{+}$ (M) and *P*${}_{k}^{-}$ (M) and the electrophilic and nucleophilic Parr functions with Hirshfeld (or CM5) atomic charges *P*${}_{k}^{+}$ (H) and *P*${}_{k}^{-}$ (H) are displayed in Table 4.

From the results for the local descriptors in Table 4, it can be concluded that C1 will be the preferred site for a nucleophilic attack and that this atom will act as an electrophilic species in a chemical reaction. In turn, it can be appreciated that C14 and C15 will be prone to electrophilic attacks and that this atomic sites will act as nucleophilic species in chemical reactions where the Blue-G1 molecule is involved.

It has been already pointed out that although condensed Fukui functions give interesting results, they are not conclusive. In particular, it has been found that when studying metallic clusters, the condensed Fukui functions can predict the results of nucleophilic and electrophilic interactions with poor reliability [105]. However, from the results obtained in our work, we can present four important considerations: (i) in the first place, it is not the same to obtain conclusions about the reliability of the condensed Fukui functions when studying metallic clusters or even solid systems than when considering pure organic molecules, as happens in our case; (ii) the reliability of the results that we have obtained is impressive because the condensed Fukui functions (and thus the dual descriptor) have been calculated using two different schemes for the partition of the electronic density (i.e., the atomic charges), and the same has been done for the Parr functions: the conclusions about the reaction sites are exactly the same; (iii) in our work, we considered and presented the calculation of the dual descriptor rather than the condensed Fukui functions, and it has been shown by Martínez-Araya that the dual descriptor is more reliable for predicting the electrophilic and nucleophilic sites than the condensed Fukui functions [106]; (iv) as we have shown in several previous works [2,3,4,5,6,7,8,9,10,11,12,13,14,15,16,17,18,19,20,21,22,23,24,25,26,27,28,29,30,31,32], the reliability of the conceptual DFT descriptors for predicting the reactive sites of a given molecular system is heavily dependent on the goodness of the model chemistry employed for the calculations where we understand for goodness the ability to fulfill the KID procedure mentioned in the Introduction section.

## 5. Conclusions

The eleven fixed RSH density functionals, which include CAM-B3LYP, LC-wPBE, M11, N12, M11L, MN12L, N12SX, MN12SX, wB97, wB97X and wB97XD, are examined to establish whether they fulfill the empirical KID procedure. The assessment is done by comparing the values from HOMO and LUMO calculations to those that the ΔSCF technique for the Blue-G1 molecule generates. This is an intermediate melanoidin pigment that is of both academic and industrial interest. The study has observed that the range-separated and hybrid meta-NGA density functionals tend to be the most suited to meeting this goal. In this case, they emerge as suitable alternative to the density functionals once it is established that the behavior of the functionals are tuned using a gap-fitting procedure. They also exhibit a desirable prospect of benefiting future studies in understanding the chemical reactivity that colored melanoidins with larger molecular weights have when reducing sugars react with proteins and peptides.

From the results of this work, it becomes evident that it is easy to predict the sites of interaction of the Blue-G1 pigment under study. This would involve having DFT-based reactivity descriptors including Parr functions and dual descriptor calculations. Evidently, the descriptors were useful in characterizing and describing the preferred reactive sites. They were also useful in comprehensively explaining the reactivity of the molecules.

Furthermore, it is also possible to predict the maximum absorption wavelength for the Blue-G1 with considerable accuracy. The prediction would involve the MN12SX density functional beginning with the HOMO-LUMO gap instead of TDDFT calculations. Such a finding is particularly crucial considering the likelihood of it being used to inform the alternative determination method on the color of larger systems such as prosthetic chromophore groups. Such becomes necessary in circumstance where it would not be possible to afford the TDDFT calculations.

Thus, it can be concluded that the model chemistry MN12SX/Def2TZVP/SMD(water) is the best combination of density functional, basis set and solvation model for the prediction of maximum absorption wavelength (starting from the KS HOMO-LUMO gap) and for the prediction of the chemical reactivity sites for the Blue-G1 melanoidin pigment, and it is the recommended methodology to follow in future studies of analog compounds.

## Figures and Tables

**Table 1:** Electronic energies of the neutral, positive and negative molecular systems (in au) of Blue-G1, the HOMO, LUMO and SOMO orbital energies (also in au), $J_{I}$, $J_{A}$, $J_{HL}$ and ΔSLdescriptors calculated with the eleven density functionals and the Def2TZVPbasis set using water as as solvent simulated with the Solvation Model Density (SMD) parametrization of the Integral Equation Formalism-Polarized Continuum Model (IEF-PCM) model.

	Eo	E+	E-	HOMO	LUMO	SOMO	J(I)	J(A)	J(HL)	ΔHL
CAM-B3LYP	−2395.4460	−2395.2561	−2395.5734	−0.2380	−0.0783	−0.1748	0.0480	0.0490	0.0686	0.0965
LC-wBPE	−2395.0237	−2394.8290	−2395.1654	−0.2812	−0.0560	−0.2234	0.0865	0.0857	0.1217	0.1674
M11	−2395.2791	−2395.0804	−2395.4158	−0.2761	−0.0603	−0.2104	0.0774	0.0764	0.1087	0.1501
M11L	−2395.2507	−2395.0467	−2395.3823	−0.1974	−0.1406	−0.1235	0.0066	0.0090	0.0112	0.0171
MN12L	−2394.3267	−2394.1354	−2394.4448	−0.1850	−0.1254	−0.1122	0.0063	0.0073	0.0097	0.0132
MN12SX	−2394.4578	−2394.2578	−2394.5853	−0.1995	−0.1271	−0.1277	0.0004	0.0004	0.0006	0.0006
N12	−2396.1437	−2395.9620	−2396.2534	−0.1732	−0.1199	−0.1013	0.0086	0.0102	0.0133	0.0186
N12SX	−2395.4309	−2395.2397	−2395.5531	−0.1926	−0.1199	−0.1239	0.0014	0.0023	0.0027	0.0040
wB97	−2396.1313	−2395.9395	−2396.2668	−0.2774	−0.0513	−0.2165	0.0856	0.0842	0.1201	0.1652
wB97X	−2395.9266	−2395.7344	−2396.0603	−0.2715	−0.0546	−0.2102	0.0793	0.0791	0.1120	0.1556
wB97XD	−2395.7877	−2395.5925	−2395.9205	−0.2619	−0.0643	−0.1995	0.0668	0.0685	0.0957	0.1353

**Table 2:** $J_{\chi }$, $J_{\eta }$, $J_{\omega }$ and $J_{CDFT}$ for the Blue-G1 intermediate melanoidin pigment.

	J${}_{\chi }$	J${}_{\eta }$	J${}_{\omega }$	J${}_{\mathrm{CDFT}}$
CAM-B3LYP	0.0005	0.0970	0.1226	0.1564
LC-wBPE	0.0004	0.1722	0.2035	0.2666
M11	0.0005	0.1538	0.1610	0.2226
M11L	0.0012	0.0156	0.0568	0.0590
MN12L	0.0005	0.0136	0.0387	0.0410
MN12SX	0.0004	0.0000	0.0009	0.0010
N12	0.0008	0.0188	0.0544	0.0575
N12SX	0.0004	0.0037	0.0101	0.0108
wB97	0.0007	0.1698	0.1783	0.2462
wB97X	0.0001	0.1583	0.1654	0.2290
wB97XD	0.0009	0.1353	0.1483	0.2008

**Table 3:** Global reactivity descriptors for the Blue-G1 intermediate melanoidin pigment calculated with the MN12SX density functional.

**Electronegativity (α)**	**Chemical Hardness (η)**	**Electrophilicity (ω)**
4.4428	1.9956	4.9457
**Electron-Donating Power ($\omega^{-}$)**	**Electron-Accepting Power ($\omega^{+}$)**	**Net Electrophilicity ($\Delta \omega^{\pm }$)**
7.1959	5.7429	12.9388

**Table 4:** The condensed dual descriptor calculated with Mulliken atomic charges Δf${}_{k}$ (M) and with NPA atomic charges Δf${}_{k}$ (N), the electrophilic and nucleophilic Parr functions with Mulliken atomic charges *P*${}_{k}^{+}$ (M) and *P*${}_{k}^{-}$ (M) and the electrophilic and nucleophilic Parr functions with Hirshfeld (or CM5) atomic charges *P*${}_{k}^{+}$ (H) and *P*${}_{k}^{-}$ (H) for the Blue-G1 molecule.

Atom	Δf${}_{k}$ (M)	Δf${}_{k}$ (N)	P${}_{k}^{+}$ (M)	P${}_{k}^{-}$ (M)	P${}_{k}^{+}$ (H)	P${}_{k}^{-}$ (H)
1C	21.86	15.00	0.4112	−0.1674	0.2228	−0.0445
2C	9.23	8.64	0.1947	−0.0404	0.1207	0.0106
3C	8.05	7.72	0.1928	−0.0218	0.1169	0.0226
4C	−6.14	−4.69	−0.0644	0.0567	−0.0054	0.0611
5C	−5.06	−3.96	−0.0548	0.0339	−0.0043	0.0489
6C	−0.26	0.01	−0.0244	−0.0181	0.0096	0.0130
7C	−0.04	−0.23	−0.0173	−0.0168	0.0079	0.0107
8C	−0.02	0.08	−0.0039	0.0003	0.0013	0.0003
9C	−0.02	0.13	−0.0029	0.0006	0.0017	−0.0001
10C	−0.02	−0.05	0.0002	0.0001	0.0002	0.0002
11C	−0.02	−0.06	−0.0003	0.0004	0.0000	0.0004
12C	−0.10	−0.04	0.0003	0.0016	0.0003	0.0014
13C	0.00	−0.01	−0.0001	0.0000	0.0001	0.0001
14C	−12.52	−9.92	−0.1063	0.2541	0.0003	0.1435
15C	−12.95	−9.52	−0.1114	0.2506	−0.0026	0.1419
16C	−0.62	−0.35	0.1831	0.1843	0.1033	0.1175
17C	−2.11	−1.73	0.1521	0.1854	0.0895	0.1199
18C	−2.66	−3.17	−0.0041	0.1051	0.0277	0.0730
19C	−3.40	−3.58	−0.0113	0.1010	0.0224	0.0744
20C	3.27	3.56	0.1269	0.0461	0.0792	0.0502
21C	1.70	2.17	0.1183	0.0710	0.0727	0.0615
22C	−0.27	−0.03	0.0048	0.0067	0.0043	0.0074
23C	−0.23	−0.01	0.0042	0.0073	0.0074	0.0108
24C	−0.17	0.03	0.0000	0.0025	0.0004	0.0025
25C	−0.02	−0.02	0.0012	0.0015	0.0009	0.0014
26C	0.16	0.06	0.0000	−0.0009	0.0012	0.0000
27C	0.26	−0.02	0.0000	−0.0004	0.0015	−0.0004
28C	−0.01	−0.01	−0.0011	−0.0012	−0.0001	−0.0002
29C	−0.02	−0.02	−0.0006	−0.0004	0.0000	0.0000
30N	1.35	1.87	0.0288	−0.0186	0.0239	0.0086
31N	1.91	1.93	0.0313	−0.0272	0.0250	0.0041
32N	−0.07	0.14	−0.0118	−0.0209	0.0061	0.0069
33N	−0.14	0.22	−0.0071	−0.0184	0.0057	0.0079
34O	−0.07	−0.11	0.0000	0.0009	0.0000	0.0009
35O	0.00	−0.01	0.0001	0.0002	0.0002	0.0002
36O	−0.11	−0.18	0.0004	0.0017	0.0005	0.0017
37O	−0.01	−0.03	0.0008	0.0012	0.0007	0.0009
38O	−0.71	−0.66	0.0017	0.0116	0.0026	0.0119
39O	−0.02	−0.03	0.0001	0.0003	0.0003	0.0005
40O	−0.07	0.02	0.0020	0.0013	0.0018	0.0014
41O	0.00	0.04	0.0001	0.0001	0.0001	0.0001
42O	0.01	0.13	0.0005	−0.0002	0.0005	−0.0002
43O	−0.02	0.04	0.0019	0.0009	0.0013	0.0005
44O	−0.02	−0.03	0.0008	0.0011	0.0006	0.0008
45O	−0.01	−0.03	0.0000	0.0000	0.0000	0.0000
46O	−0.02	−0.12	0.0002	−0.0002	0.0001	−0.0001
47O	−0.02	−0.06	0.0003	0.0007	0.0001	0.0005

## Supplementary Materials

Supplementary materials are available online.
