# Supplementary material for: Molecular Reactivity and Absorption Properties of Melanoidin Blue-G1 through Conceptual DFT
Source: Molecules. 2018 Mar 2;23(3):559. doi: 10.3390/molecules23030559 (PMC6017537; doi:10.3390/molecules23030559)
Supplement: Supplementary file 1 [file molecules-23-00559-s001.pdf]

MOLECULAR REACTIVITY AND ABSORPTION PROPERTIES OF MELANOIDIN BLUE-G1 THROUGH CONCEPTUAL DFT

SUPPLEMENTARY INFORMATION

TABLE S1. Maximum wavelength absorption (λ_{max}) of the Blue-G1 pigment calculated from the HOMO-LUMO gap and from TDDFT results in comparison with experimental value

	λ_{max}**(HL)**	**Δ(HL)**	λ_{max}**(TDDFT)**	**Δ(TDDFT)**
CAM-B3LYP	285	344	527	102
LC-wBPE	202	427	524	105
M11	211	418	523	106
M11L	802	173	559	70
MN12L	764	135	537	92
MN12SX	629	0	535	94
N12	856	227	598	31
N12SX	627	2	533	96
wB97	202	427	521	108
wB97X	210	419	526	103
wB97XD	231	398	529	100

TABLE S2. Calculated bond lengths (in Å) of the Blue-G1 intermediate melanoidin pigment with the MN12SX density functional

Bond	Distance	Bond	Distance	Bond	Distance	Bond	Distance	Bond	Distance
R(1-14)	1.389	R(6-54)	1.105	R(11-33)	1.442	R(19-33)	1.375	R(28-45)	1.319
R(1-15)	1.397	R(7-17)	1.487	R(11-63)	1.102	R(20-30)	1.350	R(29-46)	1.208
R(1-48)	1.098	R(7-23)	1.523	R(11-64)	1.103	R(21-31)	1.355	R(29-47)	1.318
R(2-14)	1.427	R(7-55)	1.102	R(12-24)	1.526	R(22-24)	1.536	R(34-73)	0.970
R(2-18)	1.375	R(7-56)	1.102	R(12-34)	1.404	R(22-36)	1.412	R(35-74)	0.969
R(2-49)	1.090	R(8-26)	1.515	R(12-65)	1.105	R(22-69)	1.108	R(36-75)	0.971
R(3-15)	1.422	R(8-30)	1.431	R(12-66)	1.112	R(23-25)	1.528	R(37-76)	0.973
R(3-19)	1.381	R(8-57)	1.105	R(13-25)	1.525	R(23-37)	1.413	R(38-77)	0.973
R(3-50)	1.090	R(8-58)	1.105	R(13-35)	1.403	R(23-70)	1.111	R(39-78)	0.969
R(4-16)	1.396	R(9-27)	1.518	R(13-67)	1.111	R(24-38)	1.406	R(41-79)	0.973
R(4-20)	1.400	R(9-31)	1.436	R(13-68)	1.105	R(24-71)	1.114	R(43-80)	0.973
R(4-51)	1.090	R(9-59)	1.105	R(14-30)	1.412	R(25-39)	1.410	R(45-81)	0.973
R(5-17)	1.395	R(9-60)	1.103	R(15-31)	1.413	R(25-72)	1.113	R(47-82)	0.990
R(5-21)	1.404	R(10-28)	1.513	R(16-32)	1.380	R(26-40)	1.205	R(37-82)	1.730
R(5-52)	1.090	R(10-32)	1.431	R(17-33)	1.381	R(26-41)	1.324	R(44-77)	1.874
R(6-16)	1.486	R(10-61)	1.105	R(18-20)	1.416	R(27-42)	1.206		
R(6-22)	1.532	R(10-62)	1.105	R(18-32)	1.375	R(27-43)	1.323		
R(6-53)	1.107	R(11-29)	1.522	R(19-21)	1.411	R(28-44)	1.209		

Table S3. Calculated bond angles (in °) of the Blue-G1 intermediate melanoidin pigment with the MN12SX density functional

**Bond**	**Angle**	**Bond**	**Angle**	**Bond**	**Angle**	**Bond**	**Angle**	**Bond**	**Angle**
A(14-1-15)	130.8	A(21-5-52)	129.6	A(8-30-14)	126.9	A(11-33-17)	129.6	A(19-21-31)	108.1
A(14-1-48)	114.5	A(5-21-19)	108.6	A(8-30-20)	123.9	A(11-33-19)	122.8	A(24-22-36)	110.7
A(1-14-2)	131.3	A(5-21-31)	143.3	A(57-8-58)	105.5	A(63-11-64)	108.4	A(24-22-69)	107.8
A(1-14-30)	120.4	A(16-6-22)	115.6	A(27-9-31)	114.8	A(24-12-34)	114.1	A(22-24-38)	110.8
A(15-1-48)	114.7	A(16-6-53)	108.3	A(27-9-59)	106.9	A(24-12-65)	107.4	A(22-24-71)	107.9
A(1-15-3)	132.1	A(16-6-54)	111.2	A(27-9-60)	107.9	A(24-12-66)	108.7	A(36-22-69)	105.8
A(1-15-31)	119.6	A(6-16-32)	121.9	A(9-27-42)	121.6	A(12-24-22)	112.3	A(22-36-75)	108.5
A(14-2-18)	105.8	A(22-6-53)	106.6	A(9-27-43)	117.8	A(12-24-38)	106.8	A(25-23-37)	109.4
A(14-2-49)	126.1	A(22-6-54)	108.5	A(31-9-59)	108.5	A(12-24-71)	107.9	A(25-23-70)	106.3
A(2-14-30)	108.3	A(6-22-24)	113.4	A(31-9-60)	112.0	A(34-12-65)	107.6	A(23-25-39)	103.9
A(18-2-49)	128.1	A(6-22-36)	109.4	A(9-31-15)	125.9	A(34-12-66)	111.9	A(23-25-72)	108.0
A(2-18-20)	109.4	A(6-22-69)	109.6	A(9-31-21)	125.8	A(12-34-73)	106.7	A(37-23-70)	109.5
A(2-18-32)	142.9	A(53-6-54)	106.0	A(59-9-60)	106.4	A(65-12-66)	106.8	A(23-37-76)	107.5
A(15-3-19)	105.7	A(17-7-23)	111.9	A(28-10-32)	113.2	A(25-13-35)	113.3	A(23-37-82)	132.4
A(15-3-50)	126.4	A(17-7-55)	111.2	A(28-10-61)	108.4	A(25-13-67)	107.7	A(38-24-71)	111.1
A(3-15-31)	108.3	A(17-7-56)	108.3	A(28-10-62)	108.5	A(25-13-68)	109.9	A(24-38-77)	108.8
A(19-3-50)	127.9	A(7-17-33)	123.0	A(10-28-44)	123.8	A(13-25-23)	115.0	A(39-25-72)	111.4
A(3-19-21)	109.7	A(23-7-55)	110.0	A(10-28-45)	116.2	A(13-25-39)	110.3	A(25-39-78)	109.4
A(3-19-33)	142.3	A(23-7-56)	108.2	A(32-10-61)	109.1	A(13-25-72)	108.2	A(40-26-41)	120.8
A(16-4-20)	105.3	A(7-23-25)	114.8	A(32-10-62)	111.7	A(35-13-67)	111.4	A(26-41-79)	112.8
A(16-4-51)	125.6	A(7-23-37)	108.4	A(10-32-16)	127.6	A(35-13-68)	107.5	A(42-27-43)	120.6
A(4-16-6)	127.4	A(7-23-70)	108.4	A(10-32-18)	124.3	A(13-35-74)	108.4	A(27-43-80)	113.1
A(4-16-32)	110.6	A(55-7-56)	107.0	A(61-10-62)	105.6	A(67-13-68)	106.8	A(44-28-45)	120.0
A(20-4-51)	129.2	A(26-8-30)	112.6	A(29-11-33)	110.5	A(14-30-20)	108.0	A(28-44-77)	136.4
A(4-20-18)	108.8	A(26-8-57)	108.5	A(29-11-63)	108.9	A(15-31-21)	108.2	A(28-45-81)	113.2
A(4-20-30)	142.7	A(26-8-58)	109.1	A(29-11-64)	108.0	A(16-32-18)	107.6	A(46-29-47)	121.2
A(17-5-21)	105.3	A(8-26-40)	123.6	A(11-29-46)	122.6	A(17-33-19)	107.5	A(29-47-82)	111.9
A(17-5-52)	125.1	A(8-26-41)	115.6	A(11-29-47)	116.1	A(20-18-32)	107.6	A(76-37-82)	105.8
A(5-17-7)	126.4	A(30-8-57)	109.0	A(33-11-63)	110.0	A(18-20-30)	108.5	A(38-77-44)	165.1
A(5-17-33)	110.6	A(30-8-58)	112.0	A(33-11-64)	111.0	A(21-19-33)	108.0	A(47-82-37)	160.8
